# Peritoneal infection after colorectal cancer surgery induces substantial alterations in postoperative protein levels: an exploratory study

**DOI:** 10.1007/s00423-024-03451-4

**Published:** 2024-08-21

**Authors:** Oskar Grahn, Klas Holmgren, Pär Jonsson, Emmy Borgmästars, Christina Lundin, Malin Sund, Martin Rutegård

**Affiliations:** 1 Department of Diagnostics and Intervention, Surgery, Umeå University, Umeå, SE-901 85 Sweden; 2 Department of Chemistry, Umeå University, Umeå, Sweden; 3 Department of Surgery, University of Helsinki and Helsinki University Hospital, Helsinki, Finland; 4 Wallenberg Centre for Molecular Medicine, Umeå University, Umeå, Sweden

**Keywords:** Anastomotic leakage, Colorectal cancer, Proteomics, Pathways, Inflammation, Recurrence, Survival

## Abstract

**Purpose:**

Peritoneal infection, due to anastomotic leakage, after resection for colorectal cancer have been shown to associate with increased cancer recurrence and mortality, as well as cardiovascsular morbidity. Alterations in circulating protein levels could help shed light on the underlying mechanisms, prompting this exploratory study of 64 patients operated for colorectal cancer with anastomosis.

**Methods:**

Thirty-two cases who suffered a postoperative peritoneal infection were matched with 32 controls who had a complication-free postoperative stay. Proteins in serum samples at their first postoperative visit and at one year after surgery were analysed using proximity extension assays and enzyme-linked immunosorbent assays. Multivariate projection methods, adjusted for multiple testing, were used to compare levels between groups, and enrichment and network analyses were performed.

**Results:**

Seventy-seven proteins, out of 270 tested, were differentially expressed at a median sampling time of 41 days postoperatively. These proteins were all normalised one year after surgery. Many of the differentially expressed top hub proteins have known involvement in cancer progression, survival, invasiveness and metastasis. Over-represented pathways were related to cardiomyopathy, cell-adhesion, extracellular matrix, phosphatidylinositol-3-kinase/Akt (PI3K-Akt) and transforming growth factor beta (TGF-β) signaling.

**Conclusion:**

These affected proteins and pathways could provide clues as to why patients with peritoneal infection might suffer increased cancer recurrence, mortality and cardiovascular morbidity.

**Supplementary Information:**

The online version contains supplementary material available at 10.1007/s00423-024-03451-4.

## Introduction

Peritoneal infection is a dreaded complication after resection for colorectal cancer. While some are isolated abscesses, most are due to anastomotic leakage. Leaks are associated with increased morbidity [1], mortality, cancer recurrence and reduced disease-free survival for both colon and rectal cancer patients [2, 3], even though the association with right-sided colon cancers remains less well-established [4]. Although the definitive mechanism linking peritoneal infection and recurrence is unknown, increasing evidence suggests that peri- and postoperative stress and inflammation might facilitate the ability of residual cancer cells to induce local as well as distant cancer recurrence by affecting angiogenic, inflammatory [5], adrenergic, immunologic and thrombotic pathways [6]. An in vitro experiment has also shown that peritoneal fluid and serum from patients with an ongoing peritoneal infection after colorectal cancer resection can stimulate colorectal cancer cell-line invasiveness [7].

Proteins associated with angiogenesis, inflammation and immune response [5, 8] may be involved in the postoperative progression of minimal residual tumour disease in colorectal cancer. Such proteins may be altered when compared to preoperative levels – some for only a few days after surgery and some for five weeks postoperatively [8]. Few studies have evaluated these and other potentially important proteins and pathways in a clinically well-characterised cohort, particularly in relation to peritoneal infection, which in turn further modulates inflammatory and immunological response.

The current exploratory study aimed to identify differentially expressed serum proteins, including top network proteins, and biological pathways in patients with peritoneal infection after colorectal cancer resection. In addition, we aimed to establish whether these alterations could be observed for a long period after surgery.

## Methods

### Study design

This is a retrospective matched cohort study. Patients who underwent elective colorectal cancer resection with a primary anastomosis at Uppsala or Umeå University hospitals between 1 January 2010 and 31 December 2015 were identified using the Swedish Colorectal Cancer Registry (SCRCR) [9]. Patients with disseminated disease at the time of resection were excluded. The SCRCR contains data on patient characteristics, perioperative data, postoperative complications including anastomotic leakage within 30 days of the primary operation, histopathological assessment and 5-year follow-up including oncological outcomes.

The registry defines rectal cancer as an adenocarcinoma with its inferior border within 15 cm of the anal verge, measured with a rigid sigmoidoscope. For this study, peritoneal infection was defined as either anastomotic leakage or intra-abdominal abscess. All patients underwent a review of medical records to verify the peritoneal infection diagnosis. We used the definition of anastomotic leakage according to the International Study Group of Rectal Cancer [10]. Cause of mortality was retrieved from the *Cause of Death Registry* for all patients who died before the end of follow-up (17 June 2021). To harmonise interpretation in the current study, registrations were reviewed to determine the seminal cause of death [11] and divided into five categories; colorectal cancer-related, cardiovascular, pneumonia or other infection, other causes, or unclassifiable.

For every case with peritoneal infection, one control with a complication-free postoperative course was selected according to pre-defined matching criteria; age (± 5 years at the primary operation), sex, tumour location (colon or rectum), pathological tumour stage (pTNM: I, II or III) and operating hospital (Uppsala or Umeå).

### Biological samples and proteins

Serum samples were requested from the Uppsala-Umeå Cancer Consortium biobank (U-CAN) [12]. The serum samples analysed in the present study were drawn at the first postoperative visit (typically 4–6 weeks after surgery), and at 1 year after surgery. Serum samples were sent to Olink Proteomics in Uppsala, Sweden for analysis. The pre-defined protein target panels “Oncology II”, “Inflammation”, and “Immune Response” (Supplementary Table 1) were used, each consisting of 92 proteins. As some proteins overlap between different panels, the number of individual proteins in these panels amounted to 266. Another four selected proteins including high-sensitivity C-reactive protein (hs-CRP), intestinal fatty-acid binding protein (I-FABP), endostatin and angiostatin were analysed using enzyme-linked immunosorbent assays (ELISA) performed in-house.

### Statistical analyses

Age and intraoperative bleeding were presented as continuous variables with interquartile ranges. The remainder were classified as categorical variables; sex (female or male), tumour location (colon or rectum), BMI (< 25 kg/m^2^, 25–30 kg/m^2^ or > 30 kg/m^2^), histopathological tumour stage (pTNM I, II or III), neoadjuvant therapy (no neoadjuvant therapy, radiotherapy or chemoradiotherapy), preoperative blood transfusion (yes or no), and defunctioning stoma (yes or no). American Society of Anesthesiologists’ (ASA) class III remained as a separate category, considering its potential association with anastomotic leakage [13, 14]. Overall survival and/or recurrence between groups were displayed with Kaplan-Meier curves and evaluated with log-rank tests.

To evaluate whether protein levels were differentially expressed, we performed orthogonal projections to latent structures effect projections (OPLS-EP) [15]. Each OPLS-EP model’s potential significance were assessed by CV-ANOVA, and a *p*-value less than 0.05 was considered statistically significant. The significant OPLS-EP models had each protein remaining in the models’ effect matrices evaluated with a paired t-test; these were controlled for multiple testing using the Benjamini-Hochberg procedure with a threshold level set at 0.10. Missing data were not included in the analyses. Tabulation of the protein quantities were expressed as mean and median fold change (i.e. the ratio between case and control).

The statistical analyses were performed using STATA version 16.1 (StataCorp, Houston, TX, USA) and MATLAB release 2017a (The MathWorks, Inc, Natick, MA, USA).

### Bioinformatics analyses

Over-representation analysis was performed using clusterProfiler [16] and ReactomePA packages in R version 4.1.1 [17]. All analysed proteins were used as background. Significantly up- or downregulated proteins were analysed separately. Over-represented Kyoto encyclopedia of genes and genomes (KEGG) [18], Reactome pathways [19], and gene ontology (GO) [20] terms were identified. A q-value of 0.10 was considered statistically significant. For network analyses, up- and downregulated proteins were analysed collectively. Protein-protein interaction (PPI) networks with a high confidence score of interactions (0.7) were retrieved from the STRING database [21]. Hub proteins, i.e. proteins with a high degree of interactions, in the PPI networks were identified using the maximal clique centrality (MCC) method in the CytoScape (version 3.8.2) plugin cytoHubba [22].

## Results

### Patient demographics and clinical data

A total of 32 cases with peritoneal infection and 32 controls with a complication-free postoperative course were included, all with samples from their first postoperative visit and 26 (81.2%) cases and 27 (84.4%) controls with samples from their one year postoperative visit (Supplementary Fig. 1).

Cases with peritoneal infection more often had higher ASA class, obesity, neoadjuvant therapy, diverting stoma, preoperative blood transfusion, increased intraoperative bleeding, longer time to both their first and second postoperative sampling when compared to controls, and less often received adjuvant chemotherapy. The timing of the cases’ first postoperative visit were slightly delayed (median 41 days) when compared to the controls’ timing (median 31 days). The seminal cause of death between groups were overall evenly matched, although there were three cases of cardiovascular mortality in the infection group compared to only one in the control group (Table 1).

**Table 1:** Clinical data for 64 colorectal cancer patients. Cases with peritoneal infection and controls with a complication-free postoperative course

Variables	Peritoneal infection	
	Not infected (*n* = 32)	Infected (*n* = 32)
**Age (years)**	67.0 (63.0–75.0)	66.0 (61.0-74.5)
**Sex**		
Male	17 (53.1%)	17 (53.1%)
Female	15 (46.9%)	15 (46.9%)
**ASA classification**		
ASA I-II	28 (87.5%)	24 (75.0%)
ASA III	4 (12.5%)	6 (18.8%)
Missing	0 (0.0%)	2 (6.3%)
**BMI (kg/m**^**2**^)		
<25	16 (50.0%)	8 (25.0%)
25–30	12 (37.5%)	15 (46.9%)
>30	4 (12.5%)	9 (28.1%)
**Preoperative transfusion**		
No	29 (90.6%)	27 (84.4%)
Yes	3 (9.4%)	5 (15.6%)
**Tumour location**		
Colon	19 (59.4%)	19 (59.4%)
Rectum	13 (40.6%)	13 (40.6%)
**Neoadjuvant treatment**		
No	24 (75.0%)	20 (62.5%)
Radiotherapy	5 (15.6%)	7 (21.9%)
Chemoradiotherapy	3 (9.4%)	5 (15.6%)
**Operating hospital**		
Uppsala	16 (50.0%)	16 (50.0%)
Umeå	16 (50.0%)	16 (50.0%)
**Diverting stoma**		
No	22 (68.8%)	19 (59.4%)
Yes	10 (31.3%)	13 (40.6%)
**Intraoperative bleeding**	200.0 (50.0-325.0)	300.0 (100.0-500.0)
**pTNM**		
I	5 (15.6%)	5 (15.6%)
II	13 (40.6%)	13 (40.6%)
III	14 (43.8%)	14 (43.8%)
**Adjuvant chemotherapy**	13 (40.6%)	10 (31.3%)
**Time to first sampling (days)**	31 (25.5–40.5)	41.0 (33–70.5)
**Time to second sampling (days)**	370 (335–391)	390 (344–444)
**Seminal cause of death**		
Colorectal cancer-related	4 (12.5%)	4 (12.5%)
Cardiovascular	1 (3.1%)	3 (9.4%)
Infection	0 (0.0%)	1 (3.1%)
Other*	1 (3.1%)	0 (0.0%)
Not classifiable	0 (0.0%)	1 (3.1%)

Patients operated with a primary anastomosis during 2010–2015. N = number; ASA = American Society of Anesthesiologists; BMI = body mass index; pTNM = pathological tumour stage. Data are presented as median (IQR) for continuous measures, and *n* (%) for categorical measures

* The only case recorded as Other was due to cancer of the gall bladder

Five cases and six controls suffered distant metastasis during their follow-up, respectively. No local recurrences were recorded and there were no discernible differences regarding recurrences or overall survival (Supplementary Fig. 2).

### Differentially expressed proteins, pathways and hub proteins

**Fig. 1:**
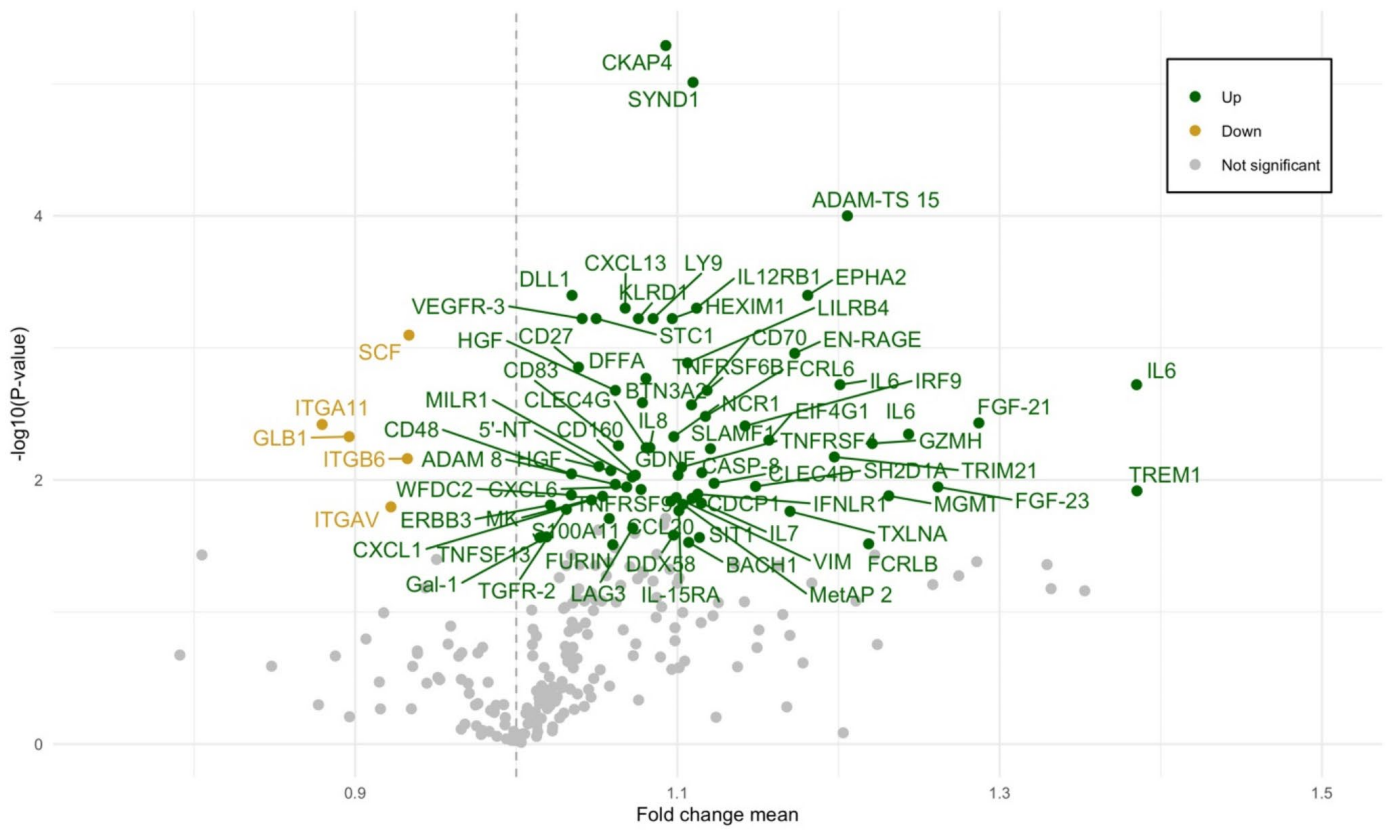
Volcano plot of evaluated proteins Differentially expressed in green (upregulated) or yellow (downregulated). One non-significant differentially expressed protein with a fold change mean of approximately 4.5 was removed from the plot to increase readability

Seventy-two proteins were upregulated and five proteins were downregulated at the first postoperative visit (Fig. 1, Supplementary Table 2).

**Fig. 2:**
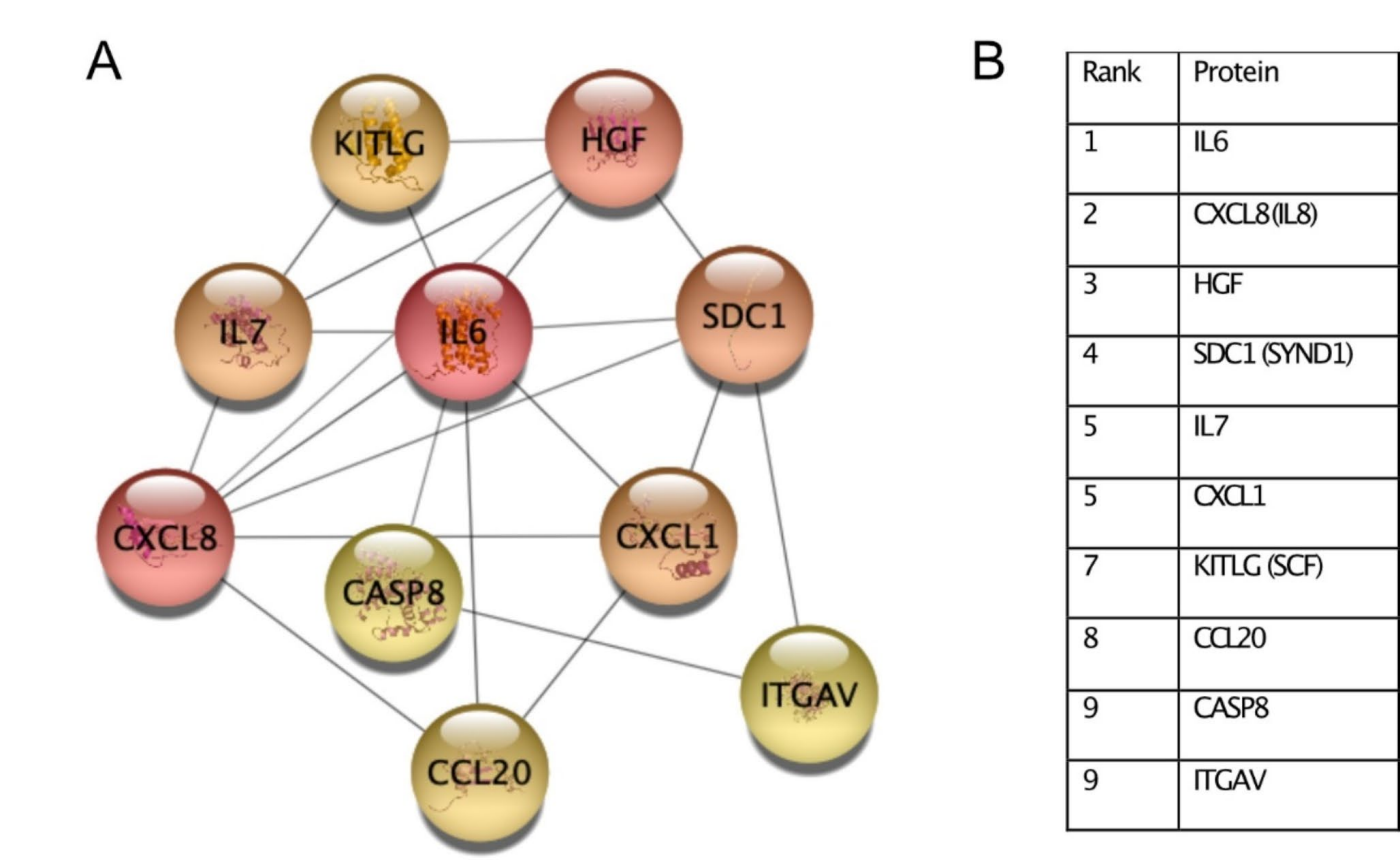
Hub proteins of differentially expressed serum proteins Top 10 ranked hub proteins were derived from differentially expressed proteins using MCC method in the CytoScape plugin cytohubba. A stronger shade of red represents a higher interconnected protein. Hub proteins are ranked in the table

Downregulated proteins were associated with pathways of cardiomyopathy, extracellular matrix (ECM), cell adhesion, phosphatidylinositol-3-kinase/Akt (PI3K-Akt) and transforming growth factor beta (TGF-β) (Supplementary Fig. 3, Supplementary Table 3). Over-representation analysis of upregulated proteins did not result in any significant pathways or GO terms. Using the MCC method in cytoHubba, the top 10 ranked hub proteins were identified (Fig. 2): IL-6, IL-8/CXCL8, HGF, SYND1/Syndecan-1, IL-7, CXCL1, KITLG/SCF, CCL20, CASP8 and ITGAV. No protein was differentially expressed to a significant degree between groups one year after surgery.

## Discussion

The aim of the present study was to explore which proteins and biological pathways are associated with postoperative peritoneal infection, and to identify essential hub proteins that may be involved in the negative consequences that can follow this complication. Patients who suffered a postoperative peritoneal infection displayed both up- and downregulation of several serum proteins related to oncological, inflammatory and immunological processes at the first postoperative visit, but with no differences in protein expression remaining after one year. There were no discernible differences regarding survival or recurrence between groups.

Several pro-angiogenic (VEGF, Ang-2, PIGF, sVCAM-1, MCP-1, MMP-3, CHI3L1, IL-8 and CXCL16) and ECM-degrading (MMP-2 and MMP-7) proteins are known to be elevated after colorectal cancer resection [8], but in relation to postoperative complications such research remain scarce. A small matched cohort study on patients with intra-abdominal infection suggested that IL-6 and VEGF were increased up to at least four days after colorectal cancer surgery [5], and that patients with infection had a substantially higher risk of cancer recurrence than controls. Moreover, peritoneal fluid and serum from patients with peritoneal infection have been shown to stimulate the invasiveness of colorectal cancer cell-lines [7], further strengthening the notion that peritoneal infection can facilitate local growth of minimal residual disease or dissemination in postoperative patients.

Several of the significantly altered hub proteins found in this study are of oncological interest. Among these, IL-6 was one of the most upregulated proteins, as well as ranked first among the hub proteins, suggesting a high degree of protein interaction. IL-6 is an important pro-inflammatory protein significantly involved in the initiation, proliferation, epithelial-to-mesenchymal transition (EMT), invasiveness and metastasis of colorectal cancer cells [23]. IL-8/C-X-C motif ligand 8 (CXCL8) is a chemokine often overexpressed in colorectal cancers, supporting migration, invasion, angiogenesis and metastasis, and has been associated with a decreased overall survival and progression-free survival [24]. Hepatocyte growth factor (HGF)/c-Met pathway plays a pivotal role in cancer invasion and metastasis, and its overexpression has been associated with colorectal liver metastasis [25]. C-X-C motif ligand 1 (CXCL1) is a growth factor exerting chemotaxis for neutrophils, for which aberrant expression is associated with colorectal cancer progression, including metastasis [26]. C-C motif ligand 20 (CCL20) performs chemotaxis for leukocytes, and is associated with cancer progression including migration and proliferation for colorectal cancer, as well as remodelling of the tumor microenvironment (TME) [27].

Furthermore, it is interesting that all of these proteins, including the upregulated inflammatory cytokine IL-6, were still differentially expressed at the first postoperative visit (median postoperative day 41) for the patients who suffered a peritoneal infection, despite a normalised hs-CRP. Thus, notwithstanding that the patients may well have recovered symptomatically and clinically, the biological perturbations caused by a peritoneal infection persist at least for a median of 41 days postoperatively, potentially still influencing adverse long-term outcomes, not least the invasiveness of minimal residual disease [7].

Many of the other differentially expressed proteins have been described in colorectal cancer progression [23, 26, 28–31], including integrins [32] of which ITGA11, ITGAV (also among the top 10 hub proteins) and ITGB6 were altered in this study. For instance, ITGA11 is overexpressed in colon cancer samples when compared to normal adjacent tissue [33], while abnormal levels of ITGAV and ITGB6 in colorectal cancer has been associated with epithelial to mesenchymal transition, cancer progression, invasiveness, metastasis [34, 35] and a worse prognosis [29, 33, 35]. Beyond cancer, integrins are also involved in atherosclerosis [36], cardiac fibrosis [37] and cardiac hypertrophy [38], and it has been shown that anastomotic leakage increases cardiovascular morbidity [1].

In the subsequent bioinformatic analyses, significant alterations in pathways related to cardiomyopathy, cell-adhesion, ECM, PI3K-Akt and TGF-β emerged. Paradoxically, these pathways were found exclusively in association to downregulated proteins, despite the numerically greater amount of upregulated proteins. Over-representation analysis in this setting is challenging considering that the involved protein panels are assembled according to biological function, and it is likely that the upregulated proteins were in such a high proportion compared to the background set that an over-representation analysis was simply not feasible. Nevertheless, alterations in pathways related to cardiomyopathy, cell-adhesion, ECM, PI3K-Akt and TGF-β were demonstrated, which could theoretically mediate increased morbidity, including cardiovascular [1], cancer recurrence [4] and mortality in patients with a postoperative peritoneal infection [4, 39].

There are several limitations to the present report. One weakness is that some important variables could not be matched due to the small sample size, including aspects of possible relevance for the study outcomes, such as neoadjuvant therapy and surgical approach. These were however considered whenever relaxation rules were applicable, i.e., when a control could be matched to several cases. Despite this, remaining bias could possibly confound our results, but we believe that our findings are probably due to the impact of peritoneal infection and not baseline differences, considering that an earlier study evaluating preoperative samples in an extended version of this cohort only saw significant differences in chemokine ligand 6 (CXCL6), CC motif chemokine ligand 11 (CCL11) and hs-CRP [40]; of these, only CXCL6 was differentially expressed in our postoperative samples, meaning that the remaining 76 differentially expressed proteins in our study were not differentially expressed preoperatively, corroborating that our findings are likely not due to confounding. Lastly, the small cohort impaired statistical power, which might be an explanation to why we could not demonstrate any influence on long-term outcomes for patients with peritoneal infection.

## Conclusion

In a context so far rarely explored, this proteomics study on patients with postoperative peritoneal infection found a possible effect on serum proteins involved in oncological processes, inflammation and immune response, as well as pathways related to cardiomyopathy, extracellular matrix organisation, cell adhesion, PI3K-Akt and TGF-β. Many of the altered hub proteins are of oncological interest including IL-6, IL-8, HGF, SYND1, CXCL1, CCL20 and ITGAV. These proteins are involved in inflammation, chemotaxis, proliferation, cell survival, epithelial-mesenchymal transition, migration, invasion and metastasis, as well as cardiovascular disease. The findings may facilitate better understanding of why overall and disease-free survival decrease, while cardiovascular complications increase, in the setting of postoperative peritoneal infection. Nevertheless, these findings need further validation in larger cohorts.

### Electronic supplementary material

Below is the link to the electronic supplementary material.

Supplementary Material 1

## Data Availability

No datasets were generated or analysed during the current study.
