# Supplementary material for: Peritoneal infection after colorectal cancer surgery induces substantial alterations in postoperative protein levels: an exploratory study
Source: Langenbecks Arch Surg. 2024 Aug 21;409(1):257. doi: 10.1007/s00423-024-03451-4 (PMC11339184; doi:10.1007/s00423-024-03451-4)
Supplement: Supplementary file 1 — Supplementary Material 1 [file 423_2024_3451_MOESM1_ESM.docx]

**Supplementary Figure 1.** **Flowchart of patient inclusion**

Potential cases, including one control each (n=116)

Patients without peritoneal infection and patients not included as controls (n=610)

Case & control without consent (n=2)

Cases & controls for whom matching was not possible (n=10)

Case and/or control missing first postoperative sample (n=40)

Remaining for analysis (n=64; 32 cases + 32 controls)

Elective surgery with a primary anastomosis for non-dissseminated colorectal cancer at Umeå or Uppsala university hospitals during 2010-2015 (n=726)

**Supplementary Figure 2**. **Kaplan-Meier curve for (A) overall survival and (B) cancer recurrence**


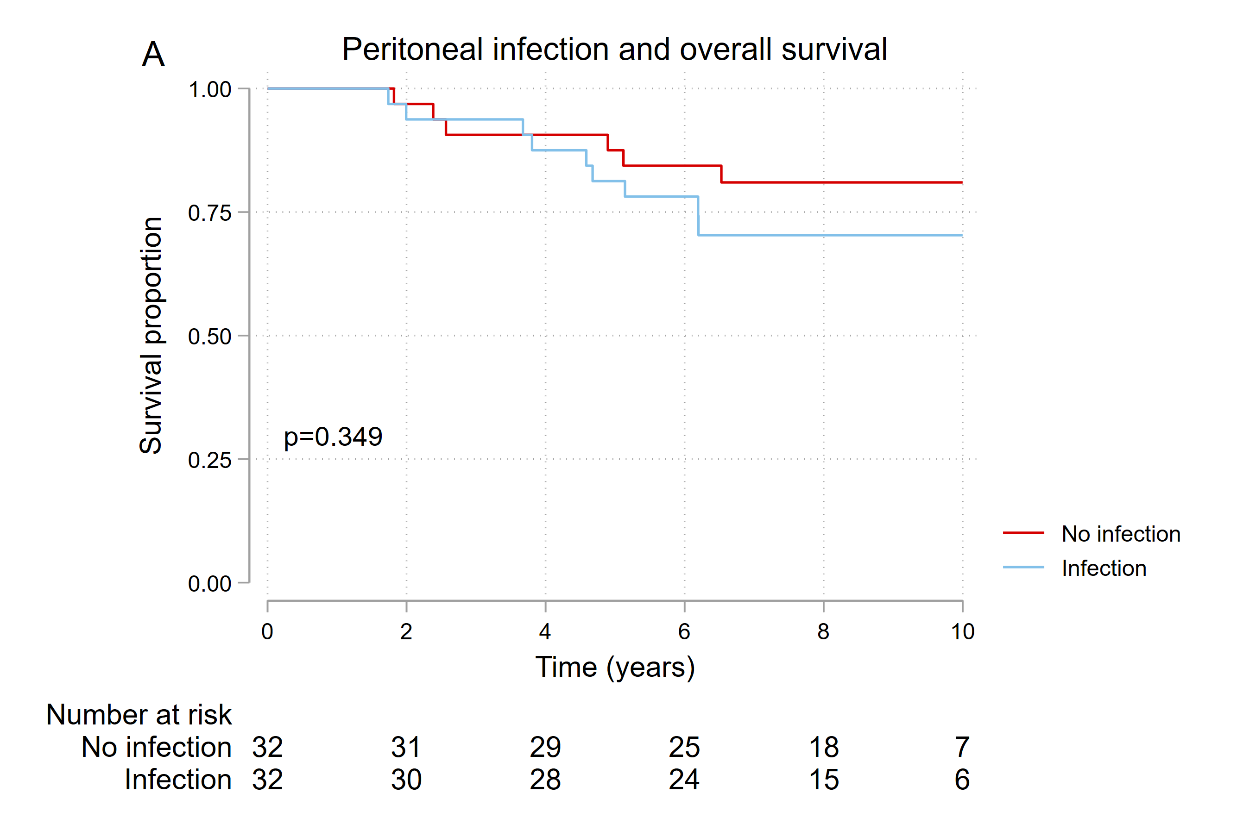

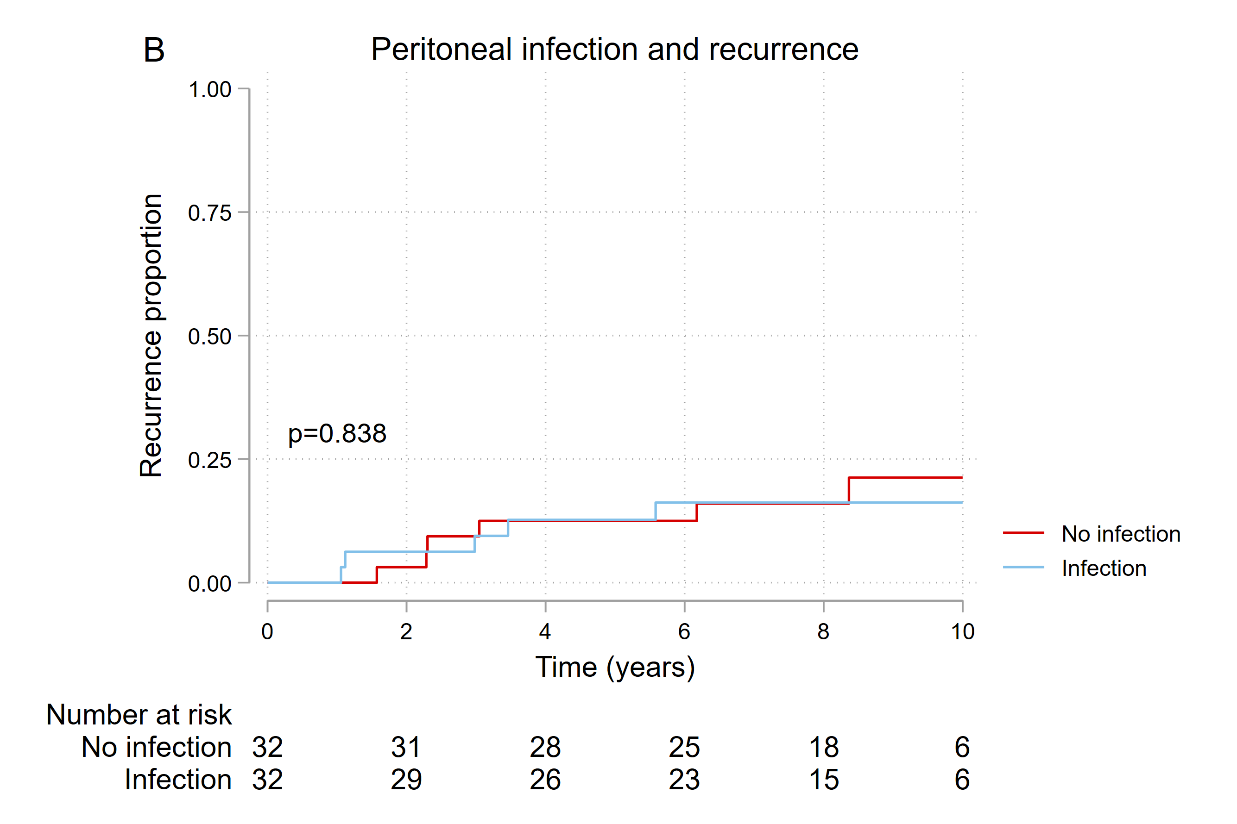


Kaplan-Meier curves for overall survival and cancer recurrence of 104 patients operated for colorectal cancer with a primary anastomosis during 2010-2015, with cases suffering peritoneal infection, contrasted to controls with a complication-free postoperative hospital stay.

**Supplementary Figure 3. Functional enrichment analysis of downregulated proteins**


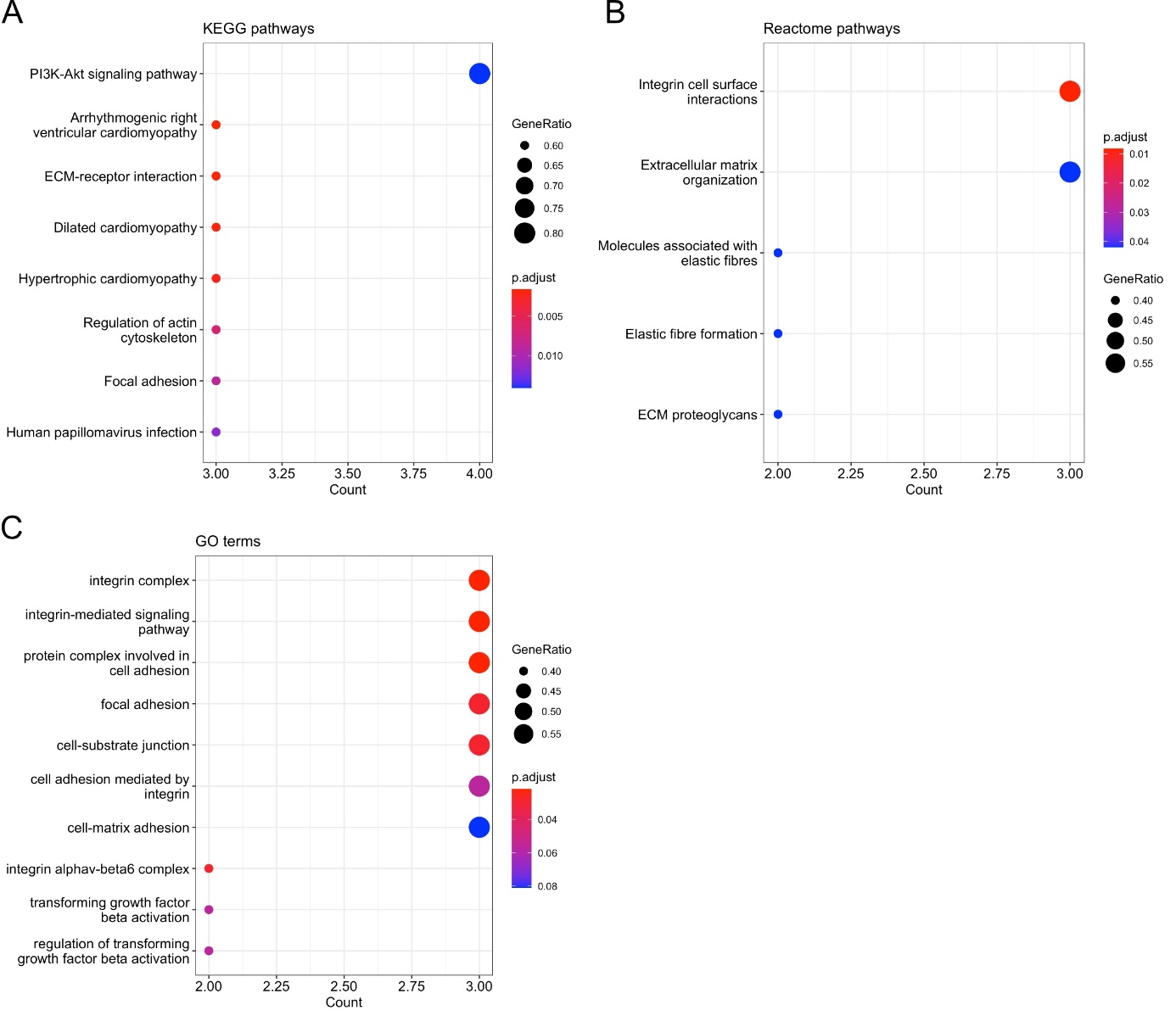


Significantly over-represented Kyoto encyclopedia of genes and genomes (KEGG) pathways (A), Reactome pathways (B), and gene ontology (GO) terms (C) that were identified. P-value adjusted for a false detection rate <0.1 using the Benjamini-Hochberg procedure. Count (x-axis) displays the number of differentially expressed proteins in each pathway. GeneRatio (circle-size) is the ratio of differentially expressed down-regulated proteins in the pathway, divided by the total amount of differentially expressed proteins that were down-regulated.

**Supplementary Table 1. Complete Olink biomarker panels**. The title of each panel is listed on the first row

| **Oncology II** | **Inflammation** | **Immune Response** |
| --- | --- | --- |
| 5'-NT | 4E-BP1 | AREG* |
| ABL1 | ADA | ARNT |
| ADAM 8 | ARTN | BACH1 |
| ADAM-TS 15 | AXIN1 | BIRC2 |
| ANXA1 | Beta-NGF | BTN3A2 |
| AREG* | CASP-8 | CCL11* |
| CAIX | CCL11* | CD28 |
| CD160 | CCL19 | CD83 |
| CD207 | CCL20 | CDSN |
| CD27 | CCL23 | CKAP4 |
| CD48 | CCL25 | CLEC4A |
| CD70 | CCL28 | CLEC4C |
| CDKN1A | CCL3 | CLEC4D |
| CEACAM1 | CCL4 | CLEC4G |
| CEACAM5 | CD244 | CLEC6A |
| CPE | CD40 | CLEC7A |
| CRNN | CD5 | CNTNAP2 |
| CTSV | CD6 | CXADR |
| CXCL13 | CD8A | CXCL12 |
| CXL17 | CDCP1 | DAPP1 |
| CYR61 | CSF-1 | DCBLD2 |
| DLL1 | CST5 | DCTN1 |
| EGF | CX3CL1 | DDX58 |
| EPHA2 | CXCL1 | DFFA |
| ERBB2 | CXCL10 | DGKZ |
| ERBB3 | CXCL11 | DPP10 |
| ERBB4 | CXCL5 | EDAR |
| ESM-1 | CXCL6 | EGLN1 |
| FADD | CXCL9 | EIF4G1 |
| FASLG | DNER | EIF5A |
| FCRLB | EN-RAGE | FAM3B |
| FGF-BP1 | FGF-19 | FCRL3 |
| FR-alpha | FGF-21 | FCRL6 |
| FR-gamma | FGF-23 | FGF2 |
| FURIN | FGF-5 | FXYD5 |
| Gal-1 | Flt3L | GALNT3 |
| GPC1 | GDNF | GLB1 |
| GPNMB | HGF* | HCLS1 |
| GZMB | IFN-gamma | HEXIM1 |
| GZMH | IL-1 alpha | HNMT |
| HGF* | IL10* | HSD11B1 |
| hK11 | IL-10RA | ICA1 |
| hK14 | IL-10RB | IFNLR1 |
| hK8 | IL-12B | IL10* |
| ICOSLG | IL13 | IL12RB1 |
| IFN-gamma-R1 | IL-15RA | IL5* |
| IGF1R | IL-17A | IL6** |
| IL6** | IL-17C | IRAK1 |
| ITGAV | IL18 | IRAK4 |
| ITGB5 | IL-18R1 | IRF9 |
| KLK13 | IL2 | ITGA11 |
| LY9 | IL-20 | ITGA6 |
| LYN | IL-20RA | ITGB6 |
| LYPD3 | IL-22 RA1 | ITM2A |
| MAD homolog 5 | IL-24 | JUN |
| MetAP 2 | IL-2RB | KLRD1 |
| MIA | IL33 | KPNA1 |
| MIC-A/B | IL4 | KRT19 |
| MK | IL5* | LAG3 |
| MSLN | IL6** | LAMP3 |
| MUC-16 | IL7 | LILRB4 |
| PODXL | IL8 | LY75 |
| PPY | LAP TGF-beta-1 | MASP1 |
| PVRL4 | LIF | MGMT |
| RET | LIF-R | MILR1 |
| RSPO3 | MCP-1 | NCR1 |
| S100A11 | MCP-2 | NF2 |
| S100A4 | MCP-3 | NFATC3 |
| SCAMP3 | MCP-4 | NTF4 |
| SCF | MMP-1 | PADI2 |
| SEZ6L | MMP-10 | PIK3AP1 |
| SPARC | NRTN | PLXNA4 |
| SYND1 | NT-3 | PPP1R9B |
| TCL1A | OPG | PRDX1 |
| TFPI-2 | OSM | PRDX3 |
| TGF-alpha* | PD-L1 | PRDX5 |
| TGFR-2 | SCF | PRKCQ |
| TLR3 | SIRT2 | PSIP1 |
| TNFRSF19 | SLAMF1 | PTH1R |
| TNFRSF4 | ST1A1 | SH2B3 |
| TNFRSF6B | STAMPB | SH2D1A |
| TNFSF13 | TGF-alpha* | SIT1 |
| TRAIL* | TNF | SPRY2 |
| TXLNA | TNFB | SRPK2 |
| VEGFA* | TNFRSF9 | STC1 |
| VEGFR-2 | TNFSF14 | TANK |
| VEGFR-3 | TRAIL* | TPSAB1 |
| VIM | TRANCE | TRAF2 |
| WFDC2 | TSLP | TREM1 |
| WIF-1 | TWEAK | TRIM21 |
| WISP-1 | uPA | TRIM5 |
| XPNPEP2 | VEGFA* | ZBTB16 |

* Biomarker represented in two panels

** Biomarker represented in three panels

**Supplementary Table 2.** **Differentially expressed proteins at the first postoperative visit.**

| Panel name / Protein name | Adjusted* p-value | Median fold change | Mean fold change |
| --- | --- | --- | --- |
| *Oncology 2 panel* |  |  |  |
| 5'-NT | 0.05 | 1.07 | 1.05 |
| ADAM 8 | 0.05 | 1.06 | 1.06 |
| ADAM-TS 15 | 0.01 | 1.12 | 1.21 |
| CD160 | 0.05 | 1.03 | 1.07 |
| CD27 | 0.01 | 1.02 | 1.04 |
| CD48 | 0.05 | 1.01 | 1.03 |
| CD70 | 0.02 | 1.14 | 1.11 |
| CXCL13 | 0.01 | 1.08 | 1.07 |
| DLL1 | 0.01 | 1.04 | 1.03 |
| EPHA2 | 0.01 | 1.16 | 1.18 |
| ERBB3 | 0.06 | 1.02 | 1.02 |
| FCRLB | 0.09 | 1.15 | 1.22 |
| FURIN | 0.09 | 1.04 | 1.06 |
| Gal-1 | 0.08 | 1.03 | 1.02 |
| GZMH | 0.03 | 1.20 | 1.22 |
| HGF** | 0.02 | 1.04 | 1.06 |
| IL6** | 0.02 | 1.50 | 1.39 |
| ITGAV* | 0.06 | 0.88 | 0.92 |
| LY9 | 0.01 | 1.08 | 1.08 |
| MetAP 2 | 0.06 | 1.07 | 1.10 |
| MK | 0.06 | 1.00 | 1.05 |
| S100A11 | 0.06 | 1.04 | 1.06 |
| SCF* | 0.01 | 0.95 | 0.93 |
| SYND1 | 0.00 | 1.12 | 1.11 |
| TGFR-2 | 0.06 | 1.02 | 1.03 |
| TNFRSF4 | 0.05 | 1.07 | 1.10 |
| TNFRSF6B | 0.02 | 1.10 | 1.12 |
| TNFSF13 | 0.08 | 1.01 | 1.01 |
| TXLNA | 0.06 | 1.25 | 1.17 |
| VEGFR-3 | 0.01 | 1.04 | 1.04 |
| VIM | 0.06 | 1.12 | 1.10 |
| WFDC2 | 0.06 | 1.02 | 1.03 |
|  |  |  |  |
| Panel name / Protein name | **Adjusted* p-value** | **Median fold change** | **Mean fold change** |
| *Inflammation panel* |  |  |  |
| CASP-8 | 0.09 | 1.06 | 1.12 |
| CCL20 | 0.09 | 1.12 | 1.10 |
| CDCP1 | 0.09 | 1.04 | 1.11 |
| CXCL1 | 0.09 | 1.03 | 1.05 |
| CXCL6 | 0.09 | 1.05 | 1.07 |
| EN-RAGE | 0.09 | 1.18 | 1.17 |
| FGF-21 | 0.09 | 1.33 | 1.29 |
| FGF-23 | 0.09 | 1.14 | 1.26 |
| GDNF | 0.09 | 1.01 | 1.10 |
| HGF** | 0.09 | 1.04 | 1.06 |
| IL-15RA | 0.10 | 1.04 | 1.10 |
| IL6** | 0.09 | 1.43 | 1.24 |
| IL7 | 0.09 | 1.08 | 1.11 |
| IL8 | 0.09 | 1.08 | 1.08 |
| SLAMF1 | 0.09 | 1.13 | 1.12 |
| TNFRSF9 | 0.09 | 1.05 | 1.08 |
|  |  |  |  |
| Panel name / Protein name | **Adjusted* p-value** | **Median fold change** | **Mean fold change** |
| *Immune Response panel* |  |  |  |
| BACH1 | 0.09 | 1.17 | 1.11 |
| BTN3A2 | 0.03 | 1.05 | 1.08 |
| CD83 | 0.03 | 1.06 | 1.06 |
| CKAP4 | 0.00 | 1.09 | 1.09 |
| CLEC4D | 0.05 | 1.16 | 1.12 |
| CLEC4G | 0.03 | 1.08 | 1.08 |
| DDX58 | 0.09 | 1.10 | 1.10 |
| DFFA | 0.02 | 1.08 | 1.08 |
| EIF4G1 | 0.03 | 1.21 | 1.16 |
| FCRL6 | 0.03 | 1.10 | 1.12 |
| GLB1* | 0.03 | 0.88 | 0.90 |
| HEXIM1 | 0.01 | 1.08 | 1.10 |
| IFNLR1 | 0.05 | 1.08 | 1.11 |
| IL12RB1 | 0.01 | 1.09 | 1.11 |
| IL6** | 0.02 | 1.22 | 1.20 |
| IRF9 | 0.03 | 1.11 | 1.14 |
| ITGA11* | 0.03 | 0.89 | 0.88 |
| ITGB6* | 0.03 | 0.92 | 0.93 |
| KLRD1 | 0.01 | 1.08 | 1.08 |
| LAG3 | 0.08 | 1.05 | 1.07 |
| LILRB4 | 0.02 | 1.14 | 1.11 |
| MGMT | 0.05 | 1.24 | 1.23 |
| MILR1 | 0.04 | 1.06 | 1.07 |
| NCR1 | 0.03 | 1.09 | 1.10 |
| SH2D1A | 0.05 | 1.07 | 1.15 |
| SIT1 | 0.09 | 1.12 | 1.11 |
| STC1 | 0.01 | 1.05 | 1.05 |
| TREM1 | 0.05 | 1.41 | 1.39 |
| TRIM21 | 0.03 | 1.12 | 1.20 |

Differentially expressed proteins for 104 patients operated for colorectal cancer with a primary anastomosis during 2010-2015 in Sweden, with cases suffering peritoneal infection and controls with a complication-free postoperative hospital stay.

* Down-regulated proteins

** Proteins overlapping between panels

**Supplementary Table 3. List of proteins** in each significant pathway, divided into sections according to database

| Database and significantly over-represented pathways | Altered proteins in each pathway |
| --- | --- |
| *Kyoto Encyclopedia of Genes and Genomes* |  |
| PI3K-Akt signaling pathway | ITGAV, ITGB6, ITGA11, SCF |
| Arrythmogenic right ventricular cardiomyopathy | ITGAV, ITGB6, ITGA11 |
| ECM-receptor interaction | ITGAV, ITGB6, ITGA11 |
| Dilated cardiomyopathy | ITGAV, ITGB6, ITGA11 |
| Hypertrophic cardiomyopathy | ITGAV, ITGB6, ITGA11 |
| Regulation of actin cytoskeleton | ITGAV, ITGB6, ITGA11 |
| Focal adhesion | ITGAV, ITGB6, ITGA11 |
| Human papillomavirus infection | ITGAV, ITGB6, ITGA11 |
|  |  |
| *Reactome* |  |
| Integrin cell surface interactions | ITGAV, ITGB6, ITGA11 |
| Extracellular matrix organisation | ITGAV, ITGB6, ITGA11 |
| Molecules associated with elastic fibres | ITGAV, ITGB6 |
| Elastic fibre formation | ITGAV, ITGB6 |
| ECM proteoglycans | ITGAV, ITGB6 |
|  |  |
| *Gene Ontology* |  |
| Integrin complex | ITGAV, ITGB6, ITGA11 |
| Integrin-mediated signaling pathway | ITGAV, ITGB6, ITGA11 |
| Protein complex involved in cell adhesion | ITGAV, ITGB6, ITGA11 |
| Focal adhesion | ITGAV, ITGB6, ITGA11 |
| Cell-substrate junction | ITGAV, ITGB6, ITGA11 |
| Cell adhesion mediated by integrin | ITGAV, ITGB6, ITGA11 |
| Cell-matrix adhesion | ITGAV, ITGB6, ITGA11 |
| Integrin alphaV-beta6 complex | ITGAV, ITGB6 |
| Transforming growth factor beta activation | ITGAV, ITGB6 |
| Regulation of transforming growth factor beta activation | ITGAV, ITGB6 |

* P-value adjusted for a false detection rate <0.1 using the Benjamini-Hochberg procedure.
